# 

**Published:** 1908-09

**Authors:** 


PUBLISHED MONTHLY. ATLANTA SUBSCRIPTION $1.00

LIBRARY S. G. O.

### JOURNAL-RECORD OF MEDICINE

Successor to { Atlanta Medical and Surgical Journal, Established 1855. and Southern Medical Record, Established 1870. } 53d Year

Vol. X. Atlanta, Ga., September, 1908. No. 6

